# A Novel Pixel-Based Spatial Modeling Framework for Mapping At-Risk Retina in Geographic Atrophy Progression

**DOI:** 10.1016/j.xops.2026.101330

**Published:** 2026-07-28

**Authors:** Liangbo Linus Shen, Yihan Bao, Gui-Shuang Ying, Tiarnan D.L. Keenan, Emily Y. Chew, Lejla Vajzovic, Jay M. Stewart, Lucian V. Del Priore

**Affiliations:** 1 Department of Ophthalmology, Duke University School of Medicine, Durham, North Carolina; 2 Department of Ophthalmology, University of California San Francisco, San Francisco, California; 3 Department of Statistics and Data Science, Yale University, New Haven, Connecticut; 4 Center for Preventive Ophthalmology and Biostatistics, Department of Ophthalmology, University of Pennsylvania, Perelman School of Medicine, Philadelphia, Pennsylvania; 5 Division of Epidemiology and Clinical Applications, National Eye Institute, National Institutes of Health, Bethesda, Maryland; 6 Department of Ophthalmology and Visual Science, Yale University School of Medicine, New Haven, Connecticut

**Keywords:** Geographic atrophy, Age-related macular degeneration, Retina imaging, Clinical trials, AREDS

## Abstract

**Purpose:**

To develop a Pixel-based At-risk Retina Mapping (PARM) framework quantifying local geographic atrophy (GA) progression in nonexudative age-related macular degeneration and evaluate its predictive performance and statistical efficiency relative to conventional area-based measures.

**Design:**

Secondary analysis of a multicenter randomized controlled trial.

**Participants:**

Eyes with GA from the Age-Related Eye Disease Study.

**Methods:**

Annual color fundus photographs were manually segmented, registered, and converted into 10-μm pixel grids. The perilesional “at-risk” zone was empirically defined as pixels with ≥5% 1-year GA conversion probability. Within this zone, 1-year GA pixel involvement was modeled using a generalized additive model with random effects for eye and participant. Predictors included pixel-level measures (distances to GA border and foveal center) and 9 eye-level factors. Prediction accuracy was evaluated using the area under the receiver operating characteristic curve (AUC). Simulation-based power analyses compared sample size requirements between the PARM framework and conventional area-based measures for detecting a 30% treatment effect (odds ratio 0.70 for pixel conversion) at 80% power (2-sided α = 0.05).

**Main Outcome Measures:**

One-year GA pixel involvement.

**Results:**

We included 239 eyes from 161 participants. Pixels within 500 μm of the GA border had ≥5% 1-year conversion risk, defining the “at-risk” region. Shorter distance to the GA border and greater distance from the foveal center were independently associated with a higher 1-year risk of GA pixel involvement (*P* < 0.001); 9 eye-level factors did not improve model fit. The pixel-based model achieved an AUC of 0.875 (95% confidence interval [CI], 0.865–0.887), outperforming the eye-level model (AUC = 0.750, 95% CI, 0.740–0.768). Detecting a 30% treatment effect required 30 eyes per arm using the PARM framework, compared with 140 eyes for square-root–transformed area and 310 eyes for total GA area.

**Conclusions:**

Pixel-based spatial modeling of GA progression within a 500-μm perilesional at-risk zone showed stronger predictive performance than conventional eye-level modeling and improved statistical efficiency relative to area-based measures in internal simulations by leveraging pixel-level distances to the GA border and foveal center. These findings support further evaluation of PARM framework for studying GA progression, with external validation required before clinical-trial implementation.

**Financial Disclosure(s):**

Proprietary or commercial disclosure may be found in the Footnotes and Disclosures at the end of this article.

Geographic atrophy (GA), the advanced form of nonexudative age-related macular degeneration, leads to irreversible loss of photoreceptors, retinal pigment epithelium, and choriocapillaris.^1^ Geographic atrophy currently affects >5 million individuals globally, with prevalence expected to rise as populations’ age.^2^ In 2023, 2 intravitreal complement inhibitors, pegcetacoplan and avacincaptad pegol, received approval from the US Food and Drug Administration for the treatment of GA, representing a major advance in age-related macular degeneration management.^3^^,^^4^ However, divergent international regulatory decisions highlight the ongoing clinical controversy regarding observation versus treatment, specifically how to weigh modest anatomic benefits against injection burdens and safety risks.^5^^,^^6^ As these agents enter clinical practice and numerous novel therapeutics undergo evaluation,^7^^,^^8^ there is a critical need for spatially resolved analytic approaches that can better characterize GA progression and support future evaluation of treatment effects.

The standard anatomical outcome measure used in GA studies, the annual rate of total GA area growth, exhibits marked interindividual variability, often differing by several-fold among participants.^3^^,^^4^^,^^9^ Differences in lesion morphology contribute substantially to this variability: multifocal, irregularly shaped, and large lesions tend to progress more rapidly than unifocal, circular, or small lesions, likely because larger multifocal lesions have greater cumulative border length adjacent to preserved retinal pigment epithelium.^10^, ^11^, ^12^, ^13^, ^14^, ^15^ The square-root transformation has been shown to reduce some of this variability and can decrease sample size requirements by approximately 50%.^10^^,^^16^, ^17^, ^18^ However, these conventional metrics fail to capture the full spectrum of GA lesion characteristics, including lesion number, perimeter, circularity, and topographic distribution of atrophy, which vary widely across patients.^10^^,^^13^^,^^19^, ^20^, ^21^ Consequently, most GA clinical trials require several 100 participants per arm to achieve adequate statistical power. Alternative anatomic metrics such as perimeter-adjusted growth rate^10^^,^^22^ and local border expansion rate^19^^,^^23^ have been proposed by us and other groups to account for morphologic variations in GA. Although these approaches represent meaningful refinements, they still do not fully encompass the spatial complexity of GA configurations or the heterogeneous locations of lesion border relative to the fovea.

In this study, we developed a Pixel-based At-risk Retina Mapping (PARM) framework to model GA progression at the level of individual image pixels. Unlike traditional eye-level or area-based analyses, this approach quantifies the probability that each pixel within a 500-μm perilesional rim will convert from uninvolved retina to image-defined GA over time. This probability represents an imaging-based measure of local GA conversion risk. The primary objective was to test the hypothesis that pixel-level conversion (“GA pixel involvement”) is driven primarily by local spatial context rather than global lesion features such as baseline area, lesion number, or perimeter. Specifically, pixels closer to existing GA borders are more likely to become involved by GA, reflecting the contiguous pattern of GA expansion, whereas pixels nearer to the fovea are less likely to convert due to the foveal sparing phenomenon and slower progression toward the fovea.^19^^,^^21^^,^^24^ Secondary objectives were to identify spatial drivers of local GA progression and to explore the statistical efficiency of this framework relative to conventional area-based measures in Age-Related Eye Disease Study (AREDS)-based internal simulations.

## Methods

### Study Population

This study analyzed individual-level data from the AREDS. The study design, protocol, and primary outcomes have been described previously.^25^ In brief, AREDS enrolled 4757 participants aged 55–80 years between 1992 and 1998 across 11 US retinal specialty clinics. Follow-up for the randomized controlled trial concluded in 2001, with an epidemiologic extension phase continuing through 2005. Participants were randomized to receive 1 of 4 oral supplement regimens, antioxidants, zinc, antioxidants plus zinc, or placebo, and underwent standardized ophthalmic examinations and stereoscopic color fundus photography at baseline and annual visits.

For this analysis, individual eye-level data were obtained from the publicly available AREDS database (dbGaP accession phs000001.v3.p1). Eyes were eligible if they met the following criteria: (1) gradable color fundus photographs at 2 visits 12 months apart; (2) measurable GA lesions entirely within the imaging field; and (3) no evidence of exudative age-related macular degeneration at either visit. Eyes with GA contiguous with peripapillary atrophy were excluded. For this analysis, the first eligible visit with gradable GA was defined as the baseline and the visit 12 months later as the 1-year follow-up.

All procedures adhered to the tenets of the Declaration of Helsinki. The AREDS protocol was approved by institutional review boards at all participating sites, and informed consent was obtained from all participants. The present secondary analysis of deidentified data was deemed exempt by the Yale University Institutional Review Board.

### Image Grading, Registration, and Image Processing

**Figure 1:**
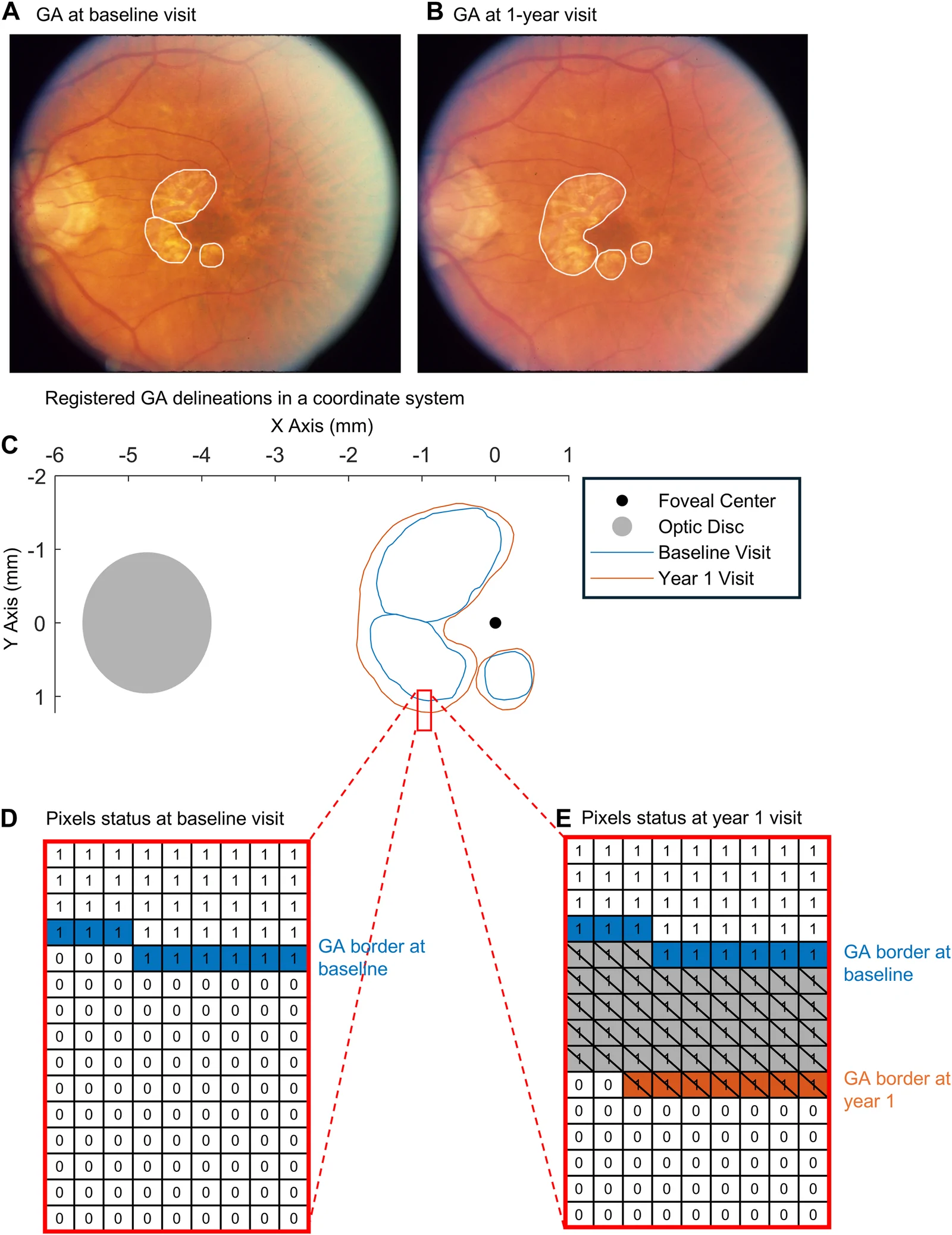
Illustration of geographic atrophy (GA) Pixel-based At-risk Retina Mapping (PARM) analysis framework. **A**, Color fundus photograph of a study eye at baseline. White outlines indicate manually delineated GA borders. **B**, The same eye at the 1-year follow-up visit. **C**, GA borders from baseline and follow-up were coregistered using three retinal vessel bifurcations and displayed in a coordinate system centered on the fovea. **D**, Pixel matrix at the baseline visit. Blue pixels indicate the GA border. Pixels within the border (pixel size = 10 × 10 μm) were assigned a value of 1 (GA-involved), and pixels outside the border were assigned a value of 0 (uninvolved). **E**, Pixel matrix at the 1-year visit. Blue pixels mark the baseline GA border, and orange pixels mark the 1-year GA border. Pixels located between the blue and orange borders represent areas that converted from non-GA to GA during follow-up (shown with diagonal lines). In the PARM analysis, the likelihood of conversion to GA was modeled for all pixels located within 500 μm of the baseline GA border.

Because the data set contained categorical gradings of GA but not quantitative lesion measurements, digitized color fundus photographs were retrieved for all eyes graded as having GA. Atrophic lesions were manually delineated using ImageJ software (version 1.52p; National Institutes of Health) following a grading protocol previously established by our group.^26^ In brief, GA lesions were manually outlined on all available visits in ImageJ. Consistent with the AREDS2 study definition, a GA lesion was required to measure at least 433 μm in diameter (corresponding to an area of 0.146 mm^2^, equivalent to one-quarter disc diameter or one-sixteenth disc area) at its widest point. The foveal center and optic disc center were marked on the baseline image, and all follow-up images were registered to baseline using a custom algorithm implemented in MATLAB (The MathWorks). Registration was based on 3 manually selected retinal vessel bifurcations and corrected for translation, rotation, and scaling differences between visits.^26^ The registered GA borders delineated in ImageJ were imported into MATLAB and displayed in a coordinate system centered on the fovea, with the optic disc–foveal axis serving as the horizontal reference (Fig 1A–C). As previously reported, this delineation and registration protocol achieved excellent reproducibility, with an intraclass correlation coefficient (ICC) of 0.997 for GA area measurements.^26^

Registration accuracy was assessed in 20 randomly selected eyes using fundus image pairs separated by 12 months in the AREDS. For each pair, 3 manually selected vessel bifurcations were used to register the follow-up image to the baseline image, and 5 additional manually identified held-out vessel bifurcations were used to calculate residual registration error. As an exploratory analysis, we also evaluated an automated deep learning-based registration workflow (EyeLiner)^27^ on the same 20 image pairs to assess and compare its registration accuracy with manual registration.

To enable spatially resolved modeling, the manually delineated GA lesions were converted into binary GA masks with a pixel resolution of 10 × 10 μm, approximately the pixel size of a fundus camera. Each pixel was assigned a value of 1 if it was involved by GA and 0 if it was uninvolved, based on the GA delineations (Fig 1D, E). Pixels already involved by GA at baseline were excluded from the progression analysis to focus on newly atrophic pixels that developed during the 1-year follow-up period. Each pixel’s distance to the nearest GA border and the foveal center was calculated using a Euclidean distance map.

### Statistical Analysis

#### Statistical Modeling of GA Pixel Involvement

All statistical analyses were performed in R software (version 4.4.3, R Foundation for Statistical Computing). Because GA expands primarily along its existing border, we first examined the spatial pattern of progression by plotting the mean percentage of pixels that developed GA over 1 year against their baseline distance from the nearest GA border. This analysis empirically defined the perilesional region where pixels had a measurable risk (≥5% annual probability) of conversion, referred to as the “at-risk” region in this paper. The primary outcome was 1-year conversion of previously uninvolved pixels within the “at-risk” region to image-defined GA.

We used a generalized additive model (GAM)^28^ to estimate the probability that an uninvolved pixel within the “at-risk” region would convert to GA over 1 year. Generalized additive models extend traditional generalized linear models by allowing nonlinear relationships between predictors and outcomes through smooth functions, providing flexibility to model spatial effects without assuming strict linearity. Spatial autocorrelation was addressed by including a 2-dimensional smooth function of pixel coordinates, and random effects were added for each eye and participant to account for clustering of pixels within eyes and between eyes of the same participant. All models were fit using the bam() function in the mgcv package in R,^28^ which is optimized for large data sets through discrete smoothing and parallel computation.

#### Identification of Factors Associated with 1-Year GA Pixel Involvement

The binary outcome variable indicated whether each previously uninvolved pixel converted to GA at the 1-year follow-up. Eleven baseline variables were evaluated as potential predictors of GA pixel involvement, including 2 pixel-level variables, distance from each pixel to the nearest GA border and distance from each pixel to the foveal center, and 9 participant-level or eye-level variables: age, baseline GA area, number of GA lesions, lesion focality (unifocal vs. multifocal), GA circularity index (GACI), lesion perimeter, foveal center point involvement, AREDS treatment group, and fellow-eye GA status. The GACI was calculated as $\frac{4\times \pi \times Total\;Area}{Total\;{Perimeter}^{2}}$.^13^ A GACI value approaching 1 indicates a more circular, compact lesion, whereas lower values reflect irregular or multifocal lesion shapes. Variable selection was performed using a forward stepwise approach guided by the Bayesian Information Criterion. Candidate predictors were sequentially added to the base model, and only variables that improved model fit by decreasing the Bayesian Information Criterion by ≥10 points were retained in the final multivariable model.

#### Model Performance Analysis

Model discrimination was evaluated using receiver operating characteristic curves and the area under the curve (AUC). Three models were compared: a pixel-based model incorporating distances from each pixel to the GA border and the foveal center; an eye-level model including age, baseline GA area, number of lesions, focality, GACI, perimeter, foveal involvement, and fellow-eye GA status; and a combined model including both predictor sets. The binary outcome in the model indicated whether each previously uninvolved pixel within the “at-risk” region converted to GA after 1 year. Model-predicted probabilities were compared with observed outcomes to generate receiver operating characteristic curves and calculate AUCs using the pROC package in R.^29^ To account for within participant and within eye pixel correlation, we calculated 95% confidence intervals (CIs) for the AUC using a cluster bootstrap approach. We generated 500 bootstrap samples by resampling at the participant level with replacement. For each iteration, we pooled all pixels from the selected participants and recalculated the AUC. The 95% CIs were empirically defined as the 2.5th and 97.5th percentiles of these 500 values. The AUC quantified each model’s ability to distinguish pixels that converted to GA from those that remained uninvolved; values >0.80 were considered strong discrimination.^30^

We further evaluated model performance using calibration plots, Brier scores, and decision curve analysis. Calibration was assessed by grouping pixels into deciles of predicted risk (10 strata), separately for each model, and plotting the observed 1-year conversion frequency against the mean predicted probability within each stratum for the eye-level, pixel-level, and all-variable models; exact 95% binomial CIs were calculated for the observed frequency in each stratum. Brier scores were calculated as the mean-squared difference between predicted probability and observed pixel conversion status, with lower values indicating better overall probability accuracy. The scaled Brier score (Brier skill score) was computed as 1 – (model Brier score divided by null-model Brier score), where the null model assigns the overall mean conversion rate to every pixel; higher values indicate greater improvement over this uninformative reference. The CIs for the Brier score were estimated using patient-level cluster bootstrap resampling with 500 resamples. Decision curve analysis was performed across threshold probabilities from 1% to 50% to compare net benefit among models and against treat-all and treat-none reference strategies.

#### Estimating Effective Sample Size in Pixel-Based Analysis

To quantify within-eye correlation among pixels, we estimated the ICC using a random-intercept logistic regression model. The model included an eye-level random intercept to account for within-eye dependence. The ICC was computed from the eye-level random-effect variance as ICC = τ^2^/(τ^2^ + π^2^/3),^31^ where τ^2^ represents the between-eye variance and π^2^/3 is the variance of the logistic distribution. To translate the ICC into the effective number of independent observations per eye, we applied the standard design-effect formula:^32^ effective sample size = m/[1 + (m – 1) × ICC], where m is the average number of pixels per eye. This calculation quantifies how pixel correlation reduces statistical independence, thereby estimating the effective amount of unique information contributed by each eye in pixel-level analysis compared with conventional eye-level (area-based) modeling.

#### Simulation-Based Power Analysis

We performed a simulation-based power analysis, following a framework similar to that described in prior literature,^33^ to estimate the number of eyes required to detect an odds ratio (OR) of 0.70 (treated vs. control) for 1-year pixel-conversion to GA (two-sided α = 0.05, target power = 0.80). For each simulated data set, eyes were randomly sampled with replacement from the AREDS study cohort, preserving their original GA lesion contours and locations, and then randomly assigned in a 1:1 ratio to treatment and control groups.

Within each sampled eye, outcomes were simulated for uninvolved pixels within the “at-risk” region. The 1-year probability of GA conversion for each pixel was modeled as the product of 4 multiplicative components: (1) the empirically derived baseline risk as a function of distance from the GA border; (2) the effect of distance to the foveal center, estimated from the GAM to account for slower progression near the fovea;^19^^,^^21^^,^^24^ (3) intereye variability, represented by multiplicative effects drawn from a log-normal distribution parameterized by the observed standard deviation (SD) of eye-specific log-odds, capturing biologic variability in progression rates; and (4) the simulated treatment effect, applied as a 30% reduction in the odds of GA conversion, corresponding to an OR of 0.70 (treated vs. control).

Two types of analyses were performed for each simulated data set. The PARM analysis was performed using the same GAM as in the primary analysis, incorporating the 2 pixel-level predictors, spatial smoothers to account for spatial correlation among neighboring pixels, and random effects to account for clustering within eyes and participants. In parallel, the area-based analysis followed conventional methods, evaluating both the total GA area growth rate (mm^2^/year) and the square-root–transformed growth rate (mm/year).^17^ For each eye, the total area of newly developed GA was calculated, and the mean change was compared between treatment and control groups. The square-root–transformed measure was further evaluated with and without adjustment for baseline covariates, including baseline GA area, lesion focality, and foveal involvement.

For each sample size, 100 independent simulations were performed to evaluate both statistical power and type I error estimation. Statistical power was defined as the proportion of simulations in which the treatment effect achieved *P* < 0.05, with sample size per arm increased incrementally until the target power of 0.80 was reached. Using the same framework, false positive rates (i.e., type 1 error rate) were estimated by setting the treatment effect to 0% (no true difference between groups); the false positive rate was defined as the proportion of simulations in which *P* < 0.05, verifying appropriate type I error control across all measures.

## Results

### Participant Characteristics and Registration Accuracy

We included a total of 239 eyes from 161 participants with GA who were followed 1 year apart in the analysis. Baseline characteristics are summarized in Table 1. The mean ± SD age of participants was 74.7 ± 6.2 years, and 57.1% were female. At baseline, the mean ± SD GA area was 5.8 ± 7.6 mm^2^, and the mean GA perimeter was 11.8 ± 10.7 mm. The mean GACI was 0.61 ± 0.30, indicating substantial variability in lesion shape and focality across eyes.

**Table 1:** Characteristics of Participants and Eyes at First Gradable GA Visit∗

Participants (Eyes), n	161 (239)
Age, years, mean (SD)	74.7 (6.2)
Sex, female, *n* (%)	92 (57.1%)
AREDS oral supplements group	
Placebo, *n* (%)	56 (23.4%)
Antioxidants, *n* (%)	55 (23.0%)
Zinc, n (%)	60 (25.1%)
Antioxidants + zinc, *n* (%)	68 (28.5%)
GA area, mm^2^, mean (SD)	5.8 (7.6)
GA perimeter, mm, mean (SD)	11.8 (10.7)
Circularity index, mean (SD)	0.61 (0.30)
Unifocal GA lesion in the eye, *n* (%)	154 (64.4%)
Number of lesions, mean (SD)	1.9 (1.7)
Center point involvement, yes, *n* (%)	69 (28.9%)
GA in the fellow eye, *n* (%)	158 (66.1%)

AREDS = Age-Related Eye Disease Study; GA = geographic atrophy; SD = standard deviation.

∗ The first eligible visit with gradable GA was defined as the baseline in the present study.

Across 20 eyes and 100 held-out validation landmark pairs, the mean ± SD residual registration error was 28.6 ± 19.9 μm, corresponding to 2.86 ± 1.99 pixels on the 10-μm analysis grid. The automated EyeLiner registration^27^ produced a similar mean ± SD residual registration error (28.5 ± 17.0 μm).

### A 500-μm Perilesional Rim Defines the “At-Risk” Region for 1-Year GA Pixel Involvement

**Figure 2:**
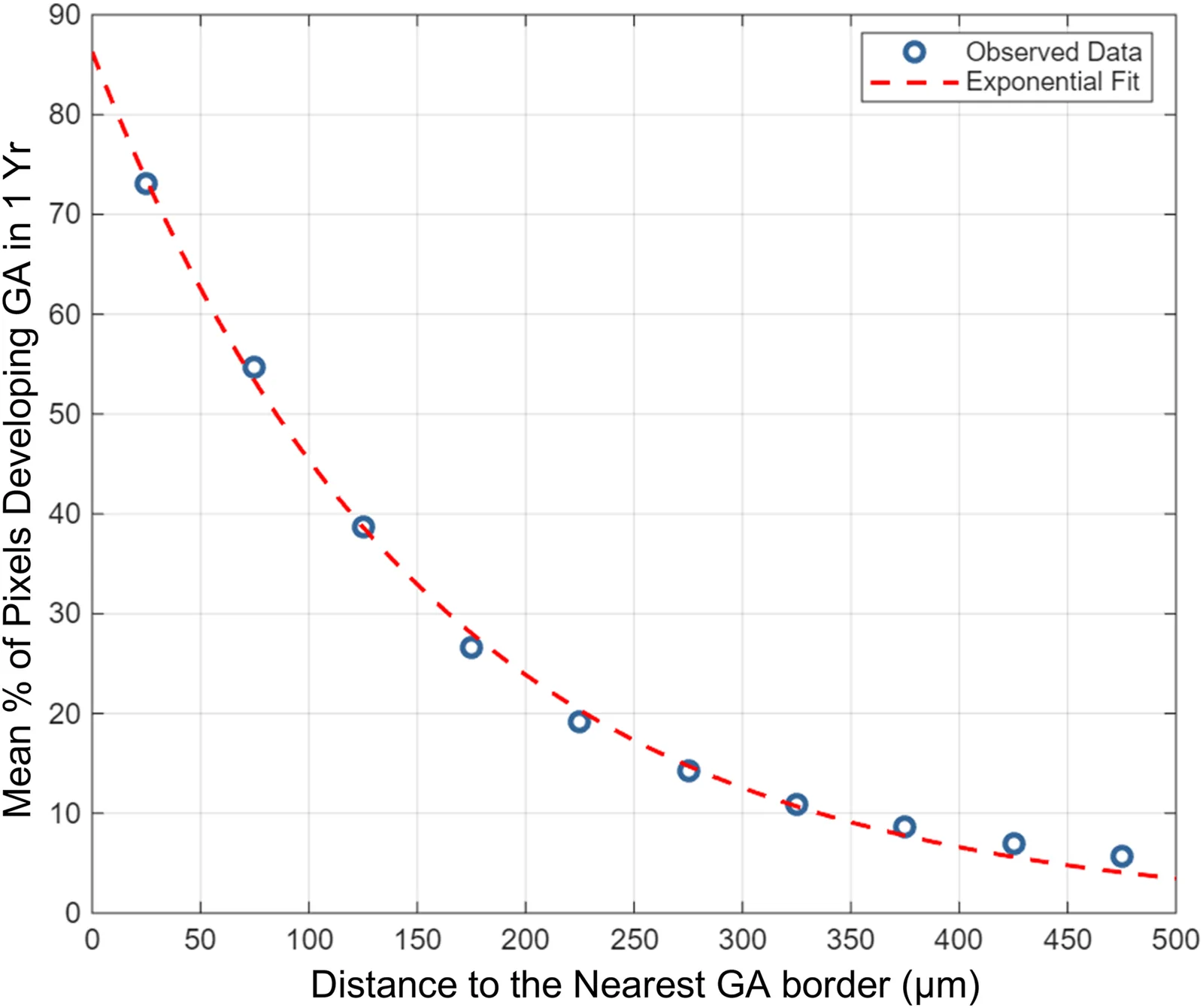
Mean percentage of pixels converting to geographic atrophy (GA) over 1 year, plotted as a function of distance from the nearest GA border. The observed data points (blue circles) were well-approximated by an exponential decay (red dashed line). Pixels within 500 μm of the GA border had at least a 5% probability of conversion over 1 year.

Geographic atrophy pixel involvement occurred predominantly along existing GA borders over 1 year (Fig 2). The mean probability of pixel conversion declined exponentially with increasing distance from the GA border. Pixels within 500 μm of the GA border demonstrated at least a 5% 1-year probability of conversion, defining the “at-risk” region used for all subsequent analyses. Beyond this distance, the 1-year likelihood of new GA involvement fell below 5%.

### Pixel Distances to GA Border and Fovea Predict 1-Year GA Involvement

A multivariable GAM with forward selection identified 2 pixel-level variables, distance to the GA border and distance to the foveal center, as the only independent predictors of 1-year GA pixel involvement. For every 100-μm increase in distance from the GA border, the odds of 1-year GA pixel involvement decreased by 65.3% (OR, 0.347; 95% CI, 0.346–0.347; *P* < 0.001). Conversely, for every 100-μm increase in distance from the foveal center, the odds of GA pixel involvement increased by 11.1% (OR, 1.111; 95% CI, 1.107–1.116; *P* < 0.001). None of the 9 participant- and eye-level variables, including age, baseline GA area, lesion number, focality, circularity index, perimeter, foveal involvement, AREDS treatment group, and fellow-eye GA, further improved model fit based on the Bayesian Information Criterion, supporting the idea that local spatial context, rather than global lesion configuration, primarily determines the risk of GA pixel involvement over 1 year.

**Figure 3:**
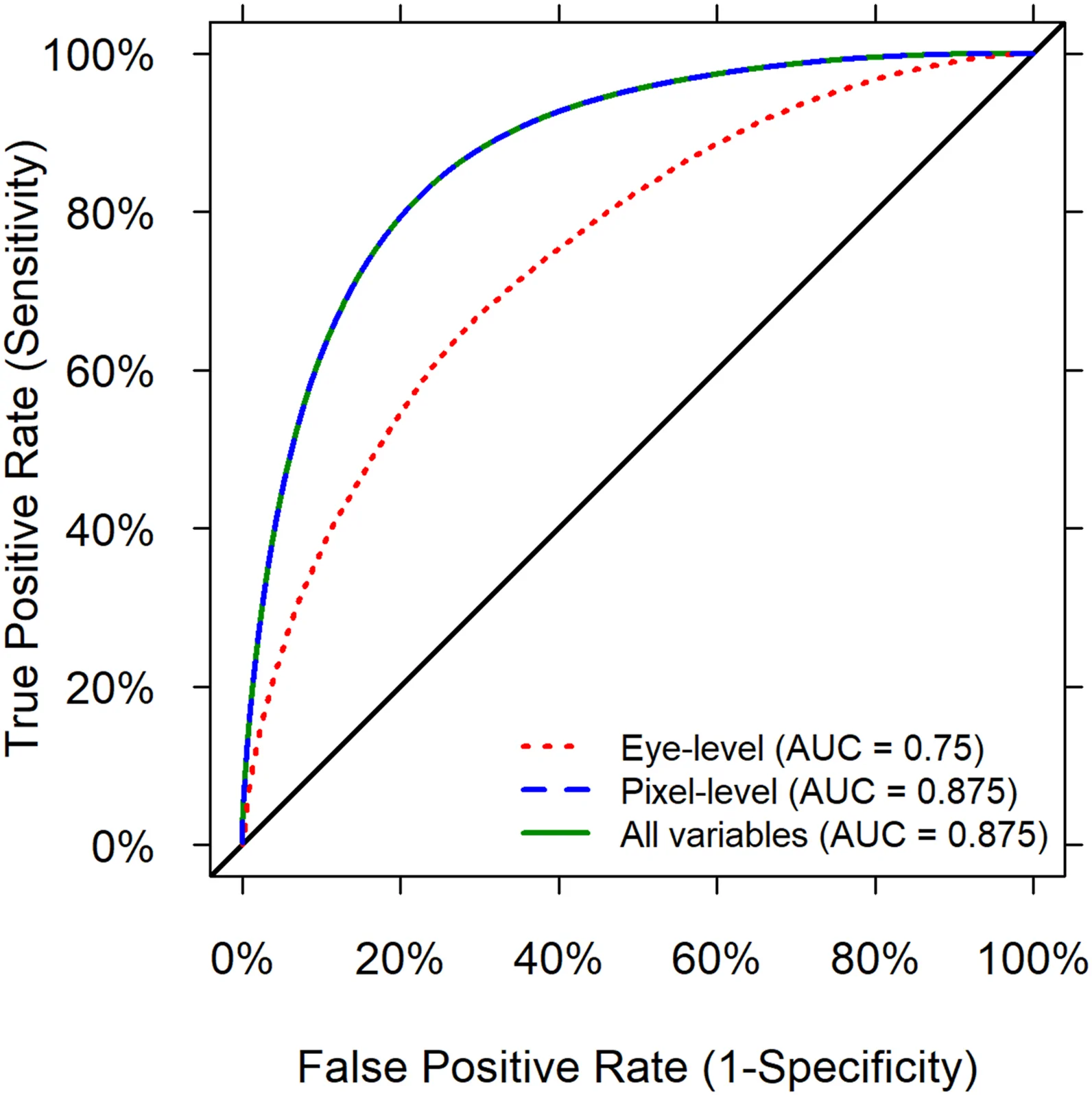
Receiver operating characteristic (ROC) curves comparing models based on pixel-level versus eye-level predictors for predicting geographic atrophy (GA) pixel involvement. A model incorporating 8 eye-level variables (red curve), including age, fellow-eye GA presence, baseline GA area, lesion number, focality, circularity index, perimeter, and foveal center involvement, achieved an area under the curve (AUC) of 0.75 (95% confidence interval [CI], 0.740–0.768). A simpler model using only 2 pixel-level variables (blue curve), specifically baseline distance from each pixel to the foveal center and to the nearest GA border, achieved a higher AUC of 0.875 (95% CI, 0.865–0.887), demonstrating superior predictive performance. Notably, adding the 8 eye-level variables to the pixel-level model did not further improve the AUC (green curve; AUC = 0.875; 95% CI, 0.865-0.887), indicating that the pixel-level features captured most of the predictive information contributed by the remaining participant- and eye-level factors in this data set.

Receiver operating characteristic analysis further demonstrated the dominant role of pixel-level predictors in forecasting GA progression (Fig 3). The pixel-level model, incorporating only each pixel’s distances to the GA border and foveal center, achieved an AUC of 0.875 (95% CI, 0.865–0.887), indicating excellent discrimination above the commonly used threshold of 0.80. In comparison, the eye-level model, which included 8 participant- or eye-level variables, achieved an AUC of 0.75 (95% CI, 0.740–0.768). Adding these eye-level predictors to the pixel-level model did not improve performance (combined AUC = 0.875; 95% CI, 0.865–0.887), confirming that the 2 pixel-level predictors captured most of the predictive information contributed by the remaining participant- and eye-level factors in this data set.

Calibration plots showed close agreement between predicted and observed 1-year pixel conversion risk across all models, indicating good apparent calibration and suggesting that predicted probabilities corresponded well to observed local GA conversion frequencies (Figure S4A, available at https://www.ophthalmologyscience.org). The pixel-level model had a lower Brier score than the eye-level model (0.120; 95% CI, 0.111–0.127 vs. 0.161; 95% CI, 0.152–0.169; Figure S4B), corresponding to greater improvement over the null model by scaled Brier score (0.372 vs. 0.157). Clinically, this indicates that the pixel-level model reduced prediction error by 37.2% compared with assigning all pixels the same average risk, versus 15.7% for the eye-level model. The all-variable model performed nearly identically to the pixel-level model, suggesting minimal incremental value from adding traditional eye-level predictors (Figure S4B). Decision curve analysis showed higher net benefit for the pixel-level and all-variable models than the eye-level model across threshold probabilities from 1% to 50% (Figure S4C), indicating better local risk stratification by identifying more pixels that subsequently became involved by GA while penalizing false-positive high-risk classifications. These findings support the PARM framework by demonstrating improved probability accuracy and potential utility for local risk stratification beyond discrimination alone.

### Pixel-Based Analysis Improves Statistical Efficiency in Internal Simulations

The mean number of pixels within the “at-risk” region was 56 940 per eye. The random–intercept logistic model yielded an ICC of 0.238, indicating moderate correlation among pixels within the same eye. Although individual pixels were not independent, each eye contributed multiple effective observations to the analysis. Using the design-effect formula, the effective number of independent pixels per eye was estimated to be 4.2, representing a 4.2-fold increase in statistical information compared with a conventional eye-level area-based analysis.

**Figure 5:**
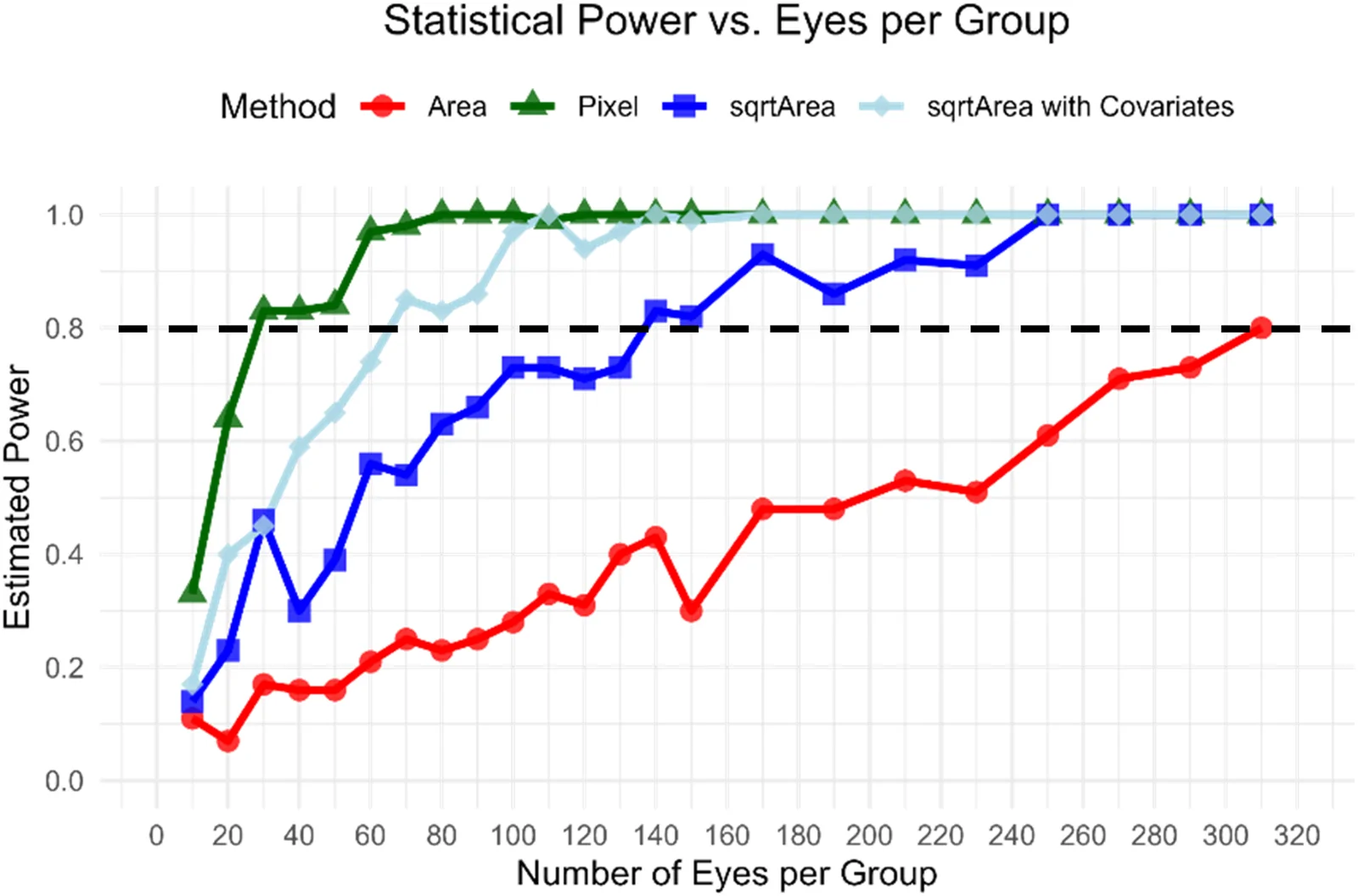
Estimated statistical power to detect a 30% treatment effect over 1 year using 4 different geographic atrophy (GA) progression measures: (1) total GA area growth rate (red), (2) square-root–transformed GA area growth rate (dark blue), (3) square-root–transformed GA area growth rate with covariate adjustment (light blue), and (4) the pixel-based method (green). Each simulation was repeated 100 times per sample size, and power was defined as the proportion of simulations achieving *P* < 0.05. The pixel-based method, which models the probability of GA conversion at the individual pixel level adjusting for each pixel’s distances to the nearest GA border and foveal center, required only 30 eyes per arm to achieve 80% power. In comparison, square-root–transformed GA with covariate adjustment required 70 eyes per arm, square-root–transformed GA alone required 140, and total GA area required 310, demonstrating improved statistical efficiency for the pixel-based framework in this cohort.

**Figure 6:**
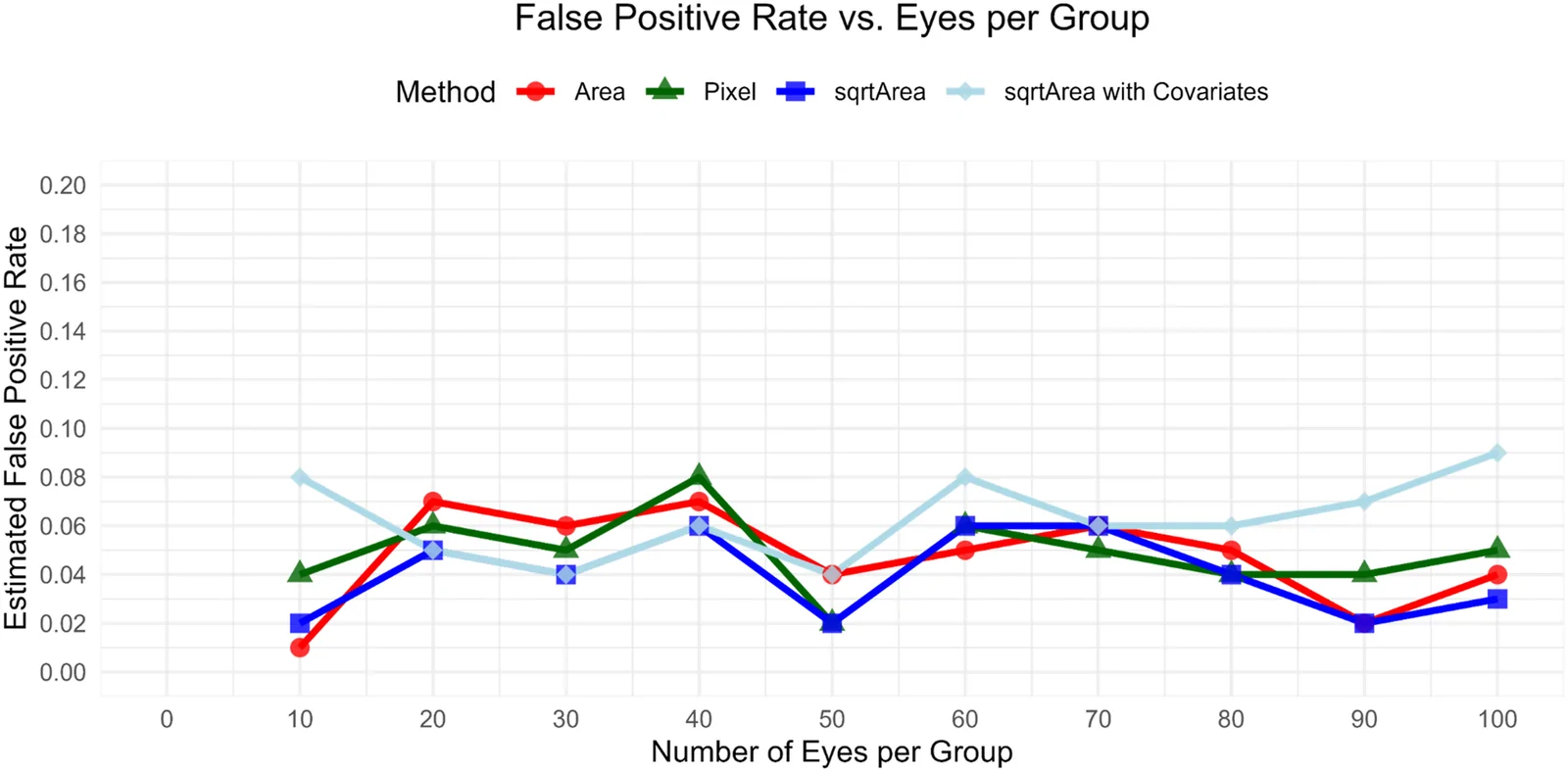
False positive rates for a 1-year geographic atrophy (GA) trial using 4 different progression measures: (1) total GA area growth rate (red), (2) square-root–transformed GA area growth rate (dark blue), (3) square-root–transformed GA area growth rate with covariate adjustment (light blue), and (4) the pixel-based method (green). Simulations were repeated 100 times per sample size, with the treatment effect set to zero (i.e., no true difference between treatment and control arms). False positive rate was defined as the proportion of simulations achieving *P* < 0.05. All 4 measures produced false positive rates near 0.05 across sample sizes, consistent with the nominal significance threshold, indicating comparable control of type I error among all metrics.

Simulation-based power analysis demonstrated that the PARM framework markedly improved statistical efficiency compared with conventional GA progression measures (Fig 5). Across 100 repeated simulations per sample size, the pixel-based method achieved 80% power with only 30 eyes per arm to detect a 30% treatment effect. In contrast, the square-root–transformed GA area method required 140 eyes per arm, and the total GA area method required 310 eyes per arm, reflecting a 10.3-fold gain in statistical efficiency. Even after adjustment for baseline covariates (GA area, focality, and foveal involvement), the square-root–transformed measure still required 70 eyes per arm, representing a 2.3-fold higher sample size compared with the pixel-based method. False positive rates remained consistent around the nominal level of 0.05 across all 4 measures (Fig 6), confirming that the PARM framework achieved substantial gains in statistical efficiency without inflating type I error.

## Discussion

In this study, we developed and internally evaluated the PARM framework for GA progression. Using GA data in 239 eyes followed 1 year apart in the AREDS, we empirically identified a 500-μm perilesional rim as the “at-risk” region, within which pixels had at least a 5% 1-year likelihood of converting to GA. A higher risk of GA pixel involvement was independently associated with shorter distance to the nearest GA border and greater distance from the foveal center. These 2 pixel-level variables captured the predictive information contained in conventional eye-level characteristics, and the pixel-level model outperformed the eye-level model in both predictive performance (AUC, 0.875 vs. 0.75) and probability accuracy with a lower Brier score (0.120 vs. 0.161). These findings suggest that prior associations between eye-level lesion characteristics and GA growth may reflect, at least in part, the spatial distribution of at-risk retina relative to the existing GA border and foveal center. Age-Related Eye Disease Study–based internal simulations suggested improved statistical efficiency relative to conventional area–based measures. These findings should be interpreted as an internal evaluation of a spatial modeling framework for GA progression, not validation of a clinical-trial endpoint. External validation in independent cohorts, contemporary imaging modalities, treated populations, and clinically meaningful outcomes is required before clinical-trial or clinic-based implementation.

### Comparison with Literature

Our results align with established imaging observations that GA enlargement occurs predominantly along existing lesion borders. A recent deep learning analysis identified the 500-μm perilesional rim as the most informative region for predicting GA progression,^34^ consistent with our empirically derived “at-risk” region threshold. Geographic atrophy is also known to expand contiguously along its existing borders,^8^^,^^35^ which explains why the distance from each uninvolved pixel to the GA border was the strongest predictor of subsequent GA pixel involvement. Consistent with this border-driven pattern, the observed probability of new GA decreased steeply with increasing distance from the baseline GA margin and followed an exponential decay pattern. This distance-dependent gradient supports the utility of local spatial context for modeling where GA is most likely to appear over 1 year.

The second key determinant, a shorter distance to the foveal center associated with a lower risk of GA pixel involvement over 1 year, also aligns with prior reports of the foveal sparing phenomenon.^24^^,^^26^^,^^35^, ^36^, ^37^ Our previous work demonstrated that local GA border expansion rates increase with retinal eccentricity within the macula,^19^^,^^21^ further supporting the protective effect of foveal proximity. Moreover, prior studies have shown that, after adjusting for GA perimeter, GA growth rate was no longer significantly associated with baseline lesion configuration (e.g., lesion area, number of lesions, or circularity),^10^^,^^22^ consistent with our current finding that eye-level lesion morphology does not independently influence pixel-level progression after adjusting for pixel-level features.

The AREDS-based internal simulation produced sample-size estimates for conventional area–based measures that were nearly identical to those obtained in our previous study of the AREDS cohort using traditional statistical methods.^10^ In that study, detecting a 30% treatment effect required 309 eyes per arm for total GA area and 141 eyes per arm for the square-root–transformed area, closely matching the 310 and 140 eyes estimated in the present simulation-based analysis. This close agreement supports the internal consistency of the simulation framework.

The PARM framework is related to, but distinct from, distance-based measures of GA border expansion. Local border expansion rate^19^^,^^23^ quantifies the linear displacement of the GA margin and is intuitive and clinically familiar, but all such metrics require an assumed point-to-point correspondence between the baseline and follow-up borders that cannot be directly observed. Different methods make different assumptions and yield different measurements: Euclidean closest-point mapping^19^^,^^23^ implies straight-line trajectories that may pass through nonatrophic retina, whereas atrophy-front growth modeling produces curved trajectories.^38^ The PARM framework avoids this ambiguity by modeling the fate of each fixed image location as a binary conversion outcome, requiring no cross-visit correspondence and remaining well defined regardless of lesion shape; spatial dependence is handled directly through the spatial smoother and random effects. In our prior AREDS analysis, GA border expansion rate using the Euclidean distance map was 0.13 ± 0.11 mm over 1 year.^19^ Based on these values, detecting a 30% reduction in eye-specific border expansion rate would require approximately 126 eyes per arm, compared with 30 eyes per arm for the PARM framework in the present AREDS-based internal simulations. This comparison provides an internal statistical-efficiency benchmark, but should not be interpreted as validation of the PARM framework as a clinical-trial endpoint.

### Mechanisms of Improved Statistical Efficiency

The reduction in required sample size observed in the AREDS-based simulations can be explained by 2 key mechanisms. First, because pixels within a single eye show only moderate correlation (ICC = 0.238), each eye contributes multiple effective observations, increasing the analytical information content approximately 4.2-fold compared with conventional eye-level analyses. The modest difference between the theoretical efficiency gain and the empirically observed gain (2.3-fold compared with the square-root–transformed model with covariate adjustment) likely reflects practical factors such as small inconsistencies in manual GA border delineation and minor registration errors between visits. Second, incorporating pixel-level spatial covariates, specifically, the distances to the GA border and foveal center, captures spatial heterogeneity in image-defined GA progression that is not represented by traditional eye-level variables. Together, these 2 factors explain the improved statistical efficiency observed in internal simulations, while underscoring that external validation is required before the framework can be used for clinical-trial decision-making.

### Potential Applications and Future Validation

The PARM framework may inform future clinical-trial design by providing a spatially resolved method to quantify GA progression. However, the present study does not establish the framework as a trial-ready endpoint. Future studies should evaluate whether aggregated pixel-level measures are reproducible across data sets, responsive to treatment effects, and meaningfully related to established GA progression measures and visual outcomes.

The framework may also reduce the influence of baseline GA morphological variability, a known contributor to variability in GA progression analyses. For instance, pegcetacoplan demonstrated a statistically significant reduction in GA growth at 12 months in the phase III OAKS trial but not in the parallel DERBY trial, whereas both achieved significance at 24 months.^3^ Post hoc analyses suggested that differences in baseline lesion characteristics between treatment arms, along with other factors such as time-dependent treatment effects and differential discontinuation, may have contributed to the early variability in efficacy outcomes, even after randomization.^39^ By modeling progression at fixed image locations, the PARM framework may better account for spatial heterogeneity in lesion morphology and location, but this potential advantage requires confirmation in independent and treated cohorts.

Although the primary analytic unit is the individual pixel, the model output can be aggregated into eye-level summary measures. The observed number or proportion of at-risk pixels converting to GA provides an eye-level measure of local progression within the at-risk zone. Summing predicted conversion probabilities across the at-risk zone and multiplying by pixel area yields an expected new GA area over 1 year. These aggregated measures may improve interpretability and facilitate comparison with conventional area-based progression metrics, but they are model-derived and require validation for reproducibility, responsiveness to treatment, and relationship to established clinical outcomes before clinical-trial use.

Beyond clinical trials, this pixel-based spatial modeling framework may eventually support clinic-based patient monitoring, but such use remains speculative. Because the framework utilizes binary atrophy masks rather than modality-specific raw measurements, it may be adaptable to GA definitions derived from fundus autofluorescence (FAF) or OCT,^8^^,^^40^ provided that lesions are segmented and spatially registered across visits. Future studies should evaluate the framework in contemporary imaging modalities, treated cohorts, and clinically meaningful outcomes before use in routine care.

### Strengths and Limitations

A major strength of this study is the introduction of a spatially resolved framework for modeling GA progression risk. The use of a large, well-characterized AREDS data set with manual lesion delineation and standardized image registration further enhances the internal consistency and reproducibility of the analyses.

However, the study has several limitations. First, although the AREDS data were collected prospectively, the present analysis was retrospective in design. Second, the framework models conversion to GA on color fundus photographs and should not be interpreted as a direct cellular measure of retinal pigment epithelium loss, photoreceptor integrity, or choriocapillaris impairment. Color fundus photographs provide lower contrast for GA boundary detection and foveal center localization accuracy compared with FAF and OCT. Because the 500-μm “at-risk” zone is defined relative to the delineated GA border, the validity of this fixed threshold depends on boundary delineation accuracy, which may vary with imaging modality. Nonetheless, multiple studies have shown strong agreement between color fundus photography and FAF or OCT imaging for measuring GA area and progression rates.^20^^,^^41^, ^42^, ^43^, ^44^, ^45^ Future validation using FAF, OCT-derived atrophy masks, and structural biomarkers will be needed to determine how image-defined pixel conversion relates to underlying biological changes. Third, because the at-risk region, model parameters, and power simulations were all derived from this single AREDS data set, the reported estimates reflect internal performance and may be subject to optimistic bias. External validation in independent cohorts, contemporary imaging modalities, treated populations, and clinically meaningful outcomes is required before clinical-trial or clinic-based implementation. Fourth, the current study did not include eyes treated with complement inhibitors. Future investigations in treated cohorts are needed to evaluate whether the PARM framework is responsive to treatment effects. Fifth, future work could incorporate additional pixel-level spatial and structural variables, such as border curvature, pixel-level directional information,^23^ local neighborhood topology (e.g., number of adjacent atrophic pixels), photoreceptor integrity,^46^^,^^47^ choriocapillaris flow deficit,^48^ or subretinal drusenoid deposits,^46^ to enhance explanatory power. Sixth, residual registration error may affect the exact correspondence of individual 10-μm pixels across visits. In our held-out landmark analysis, the mean residual error was 28.6 μm, corresponding to 2.86 pixels and only 5.7% of the 500-μm at-risk zone; automated EyeLiner registration produced a similar mean error (28.5 μm).^27^ Because neighboring pixels are spatially correlated and the main predictors vary smoothly over space, this degree of misalignment would be expected to blur local risk estimates and attenuate, rather than exaggerate, pixel-level associations. More accurate automated registration methods may further reduce spatial uncertainty and potentially improve model performance. Finally, translation of this pixel-level framework into clinical trials or clinical practice would require prespecified eye-level summary measures, standardized segmentation and registration workflows, reading-center reproducibility testing, and regulatory evaluation.

## Conclusions

In summary, this study developed and internally evaluated the PARM framework for GA progression within an empirically defined 500-μm perilesional “at-risk” zone. Pixel-level distances to the GA border and the foveal center were identified as the main determinants of 1-year GA pixel involvement in AREDS, and the PARM framework showed strong predictive accuracy and improved statistical efficiency in internal simulations. These findings support further evaluation of pixel-based spatial modeling as an analytic framework for studying GA progression. External validation in independent cohorts, contemporary imaging modalities, treated populations, and clinically meaningful outcomes is required before clinical-trial or clinic-based implementation.

## Data Availability

The data in the Age-Related Eye Disease Study (AREDS) are available in the database of Genotypes and Phenotypes (dbGaP Study Accession: phs000001.v3.p1). The raw data in our study are available on reasonable request sent to the corresponding author.

## Declaration of Generative AI and AI-Assisted Technologies in the Writing Process

During the preparation of this work, the author(s) used Gemini 3 Pro (Google LLC) and Claude (Anthropic PBC) in order to refine the writing for clarity and flow. After using this tool/service, the author(s) reviewed and edited the content as needed and take(s) full responsibility for the content of the published article.
