# Supplementary material for: A Novel Pixel-Based Spatial Modeling Framework for Mapping At-Risk Retina in Geographic Atrophy Progression
Source: Ophthalmol Sci. 2026 Jul 28;6(9):101330. doi: 10.1016/j.xops.2026.101330 (PMC13508638; doi:10.1016/j.xops.2026.101330)
Supplement: Supplementary file 1 [file mmc1.pdf]

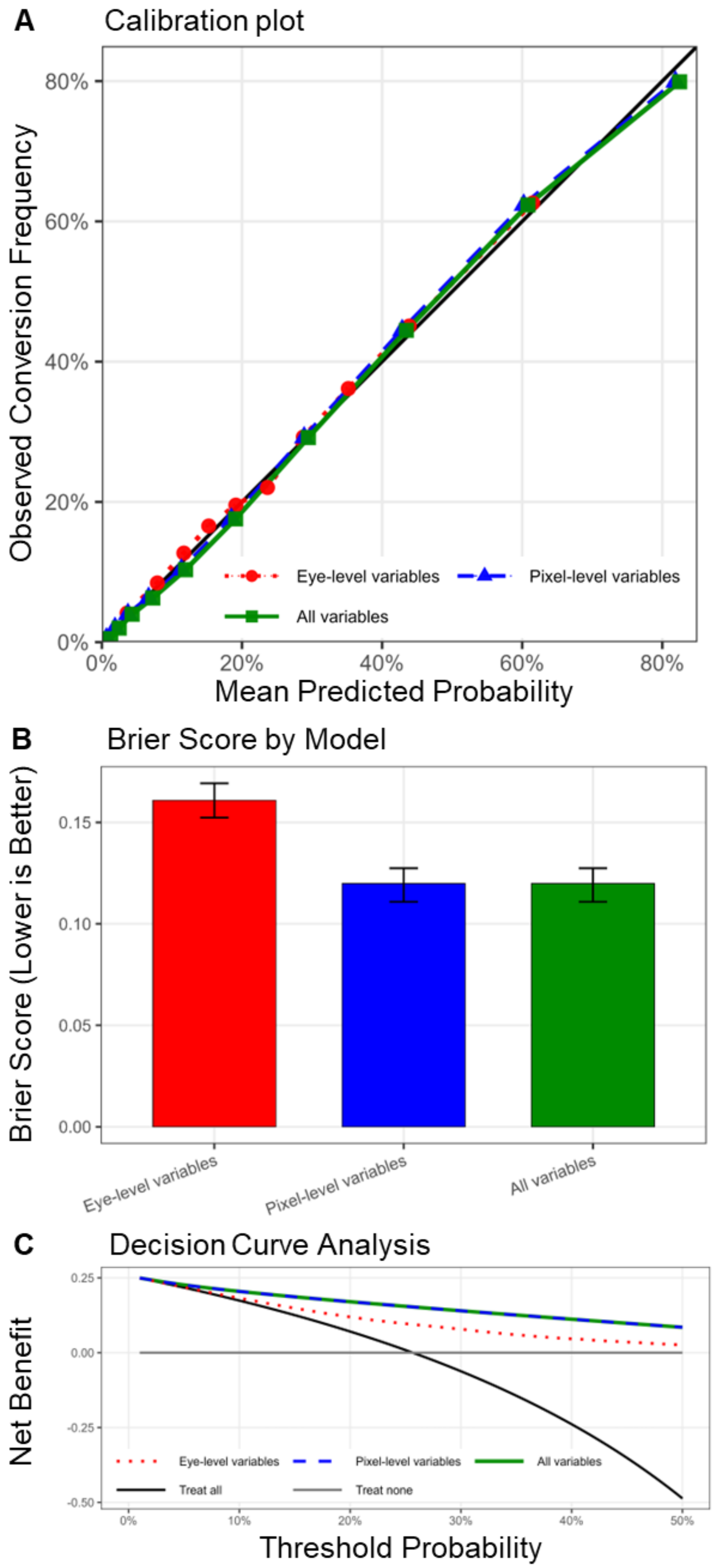

Figure S4. Calibration, Brier score, and decision curve analyses comparing pixel-level and eye-level prediction models. **A**, Calibration plot comparing mean predicted probability with observed one-year geographic atrophy (GA) pixel conversion frequency across 10 predicted-risk strata. The black diagonal line represents perfect calibration. All models showed close apparent calibration. **B,** Brier scores with 95% patient-level cluster-bootstrap confidence intervals. Lower Brier scores indicate better overall probability accuracy. The pixel-level model had a lower Brier score than the eye-level model, while the all-variable model performed nearly identically to the pixel-level model. **C,** Decision curve analysis comparing net benefit across threshold probabilities from 1% to 50%. The pixel-level and all-variable models showed higher net benefit than the eye-level model across the evaluated threshold range, supporting improved local GA risk stratification with the pixel-based framework.
